# Prior Design for Dependent Dirichlet Processes: An Application to Marathon Modeling

**DOI:** 10.1371/journal.pone.0147402

**Published:** 2016-01-28

**Authors:** Melanie F. Pradier, Francisco J. R. Ruiz, Fernando Perez-Cruz

**Affiliations:** 1 Signal Theory and Communications Department, University Carlos III in Madrid, Madrid, Spain; 2 Gregorio Marañón Health Research Institute, Madrid, Spain; 3 Data Science Institute, Columbia University, New York, United States of America; 4 Member of the Technical Staff at Bell Labs, Alcatel-Lucent, Murray Hill, New Jersey, United States of America; University of Louisville, UNITED STATES

## Abstract

This paper presents a novel application of Bayesian nonparametrics (BNP) for marathon data modeling. We make use of two well-known BNP priors, the single-p dependent Dirichlet process and the hierarchical Dirichlet process, in order to address two different problems. First, we study the impact of age, gender and environment on the runners’ performance. We derive a fair grading method that allows direct comparison of runners regardless of their age and gender. Unlike current grading systems, our approach is based not only on top world records, but on the performances of all runners. The presented methodology for comparison of densities can be adopted in many other applications straightforwardly, providing an interesting perspective to build dependent Dirichlet processes. Second, we analyze the running patterns of the marathoners in time, obtaining information that can be valuable for training purposes. We also show that these running patterns can be used to predict finishing time given intermediate interval measurements. We apply our models to New York City, Boston and London marathons.

## 1 Introduction

Data in the real world typically involves some source of uncertainty. This uncertainty may come from noisy measurements, incomplete information, or from the fact that we only have access to a subset of the data from a larger population. Probabilistic models have proven to be an effective approach for understanding such data, by incorporating our assumptions and prior knowledge of the world. In fact, probabilistic models have become an important tool in all areas of science as a way to develop statistical algorithms that are able to learn hidden structures from the data and make predictions [1].

Within probabilistic models, Bayesian nonparametric (BNP) models present several desirable properties [2]. Their most recognizable benefit is to avoid the need of specifying a closed-form model, i.e., a parametric model with a predefined fixed number of hidden variables. Instead, BNP priors place probability mass on an infinite range of models and let the inference procedure choose the one that best fits the data, in order to provide competitive predictions or density estimations [3].

Although this flexibility makes such models attractive for experts in other fields, obtaining interpretable results might be an even stronger requirement. Most BNP models are described as general priors [4–7] that might not give easy-to-interpret solutions if applied blindly, even if they provide accurate predictions. In order to additionally obtain interpretable results, we should tailor the design of the model and include all our prior knowledge in the assumptions. We need to take problem-specific design choices that will allow for an easy interpretation of the results afterwards.

In this way, the first insights of the obtained results should not be foreign to us. This makes the model trustworthy for experts in other fields that do not know about machine learning or statistics, so other conclusions that were not common knowledge can be taken as plausible. At this stage we are able to formulate hypotheses that can be tested with future data and can provide previously unknown insights about the given problem. Such procedure avoids the frequent black-box flavor found in other methods, facilitating collaboration across fields. Examples of such multi-disciplinar efforts can be found in psychiatry [8], genetics [9], biostatistics [10], computer vision [11], econometry [12] or musicology [13].

This paper presents a novel application of BNPs to model marathon runners. We opt for BNP models to leave room for the unexpected. In particular, we make use of the dependent Dirichlet process (DDP) [14], which is a powerful tool that encompasses the Dirichlet process (DP) and the hierarchical Dirichlet process (HDP). However, the DDP is very general and it cannot be directly applied to data without additional constraints. Here, we specify a way to tie the parameters across groups using a Gaussian process [15], thus making the DDP a practical prior for our problem at hand.

This paper is an application of the single-p DDP and the HDP to marathon modeling. The novelty of the paper relies not only on the application, but also on the necessary steps to transform a prior that provides accurate estimates into a prior that also gives interpretable results. Non-trivial structural assumptions and design solutions are made to find hidden properties of the athletes while providing accurate predictions. We believe that BNP models will be more useful in the future for experts without machine learning expertise if we can tailor the priors to provide accurate and understandable solutions.

The rest of the paper is organized as follows. Section 2 describes the two problems addressed in this paper. In Section 3, we review the BNP priors behind our models. We then introduce the statistical models used in this paper. In particular, Section 4 presents the atom-dependent Dirichlet process (ADDP), a collection of BNP mixture models based on the single-p DDP prior coupled with a Gaussian process, while Section 5 describes an HDP over probability vectors to detect latent running profiles and perform predictions. Section 6 is devoted to the experiments and results. Our conclusions and further discussion can be found in Section 7.

## 2 Problem Overview

### 2.1 Age-grading problem

We first consider the age-grading problem, in which we want to compare fairly the finishing times of runners having different age and gender. Currently, most popular marathons award entry to participants by their best marathon in the previous 12 months. For example, in Boston it is the only way a participant can gain entry to the race, while other paths are available in New York, Chicago or London. The proposed methodology can be used to equalize entry requirements for different marathons, which vary considerably for one event to the next, as there is no widely accepted standard method to specify them. Furthermore, the World Master Athletics (WMA) has an age-grading system [16] for equalizing the finishing time according to age and sex. They lobby for this measure to be taken into consideration for selecting the winners of each race, even though that procedure is based on world records, i.e., outliers that may not be very representative, or even realistic, for most races. Our method also provides an alternative way to reward runners of different ages and gender.

Our approach consists in adapting a single-p DDP [5] to cluster the finishing time for each runner according to his/her age and sex. We propose a Gaussian process to control how the clusters (representing marathon finishing time) change from one group to the next (different ages or gender). We find that the means of these clusters are directly comparable to the marathon entry requirements and the age-graded tables from the WMA. Additionally, direct comparisons for any finishing time are straightforward, since we find a full distribution for all ages and both genders. Our single-p DDP can simultaneously deal with different races and/or the same race on different years, providing a unified ranking for all the races that may differ on elevation profile, temperature or humidity.

### 2.2 Running pattern problem

The second problem considered herein is the temporal analysis of running patterns. Our objective here is twofold. First, we are interested in a tool that can capture latent running profiles that reflect the marathon difficulties along the 26.2 miles (42.195 km). This can be useful for athletes training purposes. Second, we aim at predicting the arrival time of runners using intermediate records. This problem has already been addressed in [17], where the finishing times are imputed for the 2013 Boston marathon. One of the best approaches rely on the 100 nearest neighbors, which has the limitation of clustering runners that are doing the same absolute times.

We propose to use an HDP [6] to model the fraction of time each runner has spent at each intermediate interval (typically, measures are taken every 5 km and at half-marathon). In this way, we cluster the time ratio instead of the absolute times. Runners that run at different but constant speed will be in the same cluster, no matter if they run each mile in five, eight or eleven minutes. Thus, this model allows estimating finishing times for slower runners that have the same time-ratio profile than fast ones. We use an HDP model in which the likelihood function is a Dirichlet distribution, and each DP clusters the runners by age group and sex. When modeling the full race, it helps to understand the different trade-offs and which parts of the race are harder.

## 3 Dependent Dirichlet processes

In this Section, we briefly review the stochastic processes on which our models are based. In particular, we focus on the DDP and two of its variants. The DDP is a generalization of the DP for groups of data [5]. The DP is a stochastic process whose realizations are random infinite discrete probability distributions [18]. A DP is completely specified by a base distribution *H*, which is the expected value of the process, and a positive real number *α* (usually referred to as concentration parameter), which plays the role of an inverse variance. In general, a draw *G*_0_ ∼ DP(*α*, *H*) from a DP can be expressed as

$$G_{0}=\sum_{k=1}^{\infty }\pi_{k}\delta_{\phi_{k}},$$ (1)

where the vector *π* contains the atom weights, and *ϕ*_*k*_ are the atom locations defined in the parameter space. In this form, it can be shown that the atom locations *ϕ*_*k*_ are independent and identically distributed as *ϕ*_*k*_ ∼ *H*, whilst the weights (also known as stick proportions) are distributed as *π* ∼ GEM(*α*), a distribution named after Griffiths, Engen and McCloskey [19, 20]. Such representation is called stick-breaking representation [21].

Note that Eq (1) defines an infinite mixture model, i.e., a mixture model with a countably infinite number of clusters. However, since the weights *π*_*k*_ decrease exponentially fast, only a finite number of clusters are used to describe any finite dataset [3]. In fact, the expected number of components grows logarithmically with the number of observations [6]. In the DP mixture model, the actual number of clusters describing the data is not fixed, and can be automatically inferred from the data using the usual Bayesian posterior inference framework, e.g., Markov chain Monte Carlo [22] or variational [23] methods.

The Chinese restaurant process (CRP) is typically found in the literature of DPs, a standard culinary metaphor that vividly illustrates how the DP operates [6]. In this metaphor, the atom locations *ϕ*_*k*_ are referred to as “dishes” in a restaurant, and observations that are clustered together are viewed as customers sitting on the same table, therefore eating from the same dish. In this generative process, customers enter the restaurant one at a time, and they can either sit on an existing table, with probability proportional to the number of previous customers that are already sitting on that table, or open a new table, with probability proportional to *α*. In the latter case, they also sample a new dish from the prior, i.e., *ϕ*_*k*^new^_ ∼ *H*.

**Fig 1:**
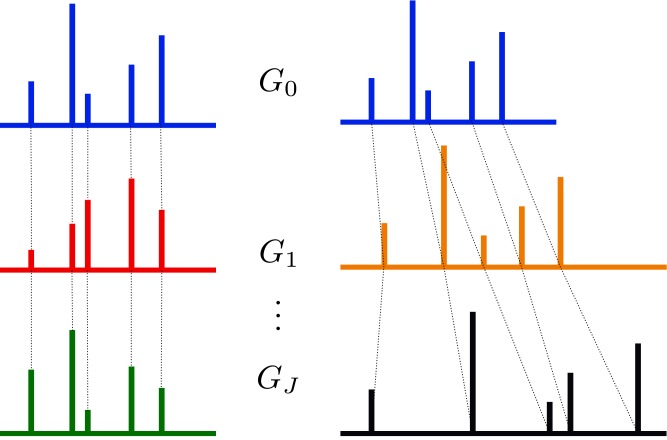
Comparison of two Dependent Dirichlet processes. Hierarchical DP is at the left, single-p DDP is at the right. The first one shares atom locations, while the second one shares mixture weights.

In its more general form, the DDP is a stochastic process that generalizes the DP and that can be applied for clustering groups of data [5]. For each group *j*, we have an infinite mixture model of the form

$$G_{j}=\sum_{k=1}^{\infty }\pi_{jk}\delta_{\phi_{jk}},$$ (2)

where *G*_*l*_ is a group-specific random measure, and the atom weights *π*_*jk*_ and atom locations *ϕ*_*jk*_ follow stochastic processes on the covariate space *j*. We describe below the two particularizations of the DDP that we use throughout the paper: the hierarchical DP (HDP) and the single-p DDP. Fig 1 shows a comparative sketch between both models.

### 3.1 Hierarchical Dirichlet process

The HDP is a particular DDP to cluster groups of data sharing mixture components [6]. It uses a DP for each group of data, with the DPs for all groups sharing a base distribution which is itself drawn from a DP. Mathematically, we first draw a base distribution from a DP as *G*_0_ ∼ DP(*γ*, *H*), where $G_{0}=\sum_{k=1}^{\infty }v_{k}\delta_{\phi_{k}}$, and for each group *j* we draw a distribution from a DP using *G*_0_ as the base distribution, i.e., *G*_*j*_ ∼ DP(*α*, *G*_0_). This construction ensures that all the distributions *G*_*j*_ share the atom locations given by *G*_0_, since *G*_0_ is itself a discrete probability distribution. Since each atom corresponds to a cluster, cluster parameters *ϕ*_*k*_ are shared across all groups. Each *G*_*j*_ admits a representation as

$$G_{j}=\sum_{k=1}^{\infty }\pi_{jk}\delta_{\phi_{k}},$$ (3)

where the atom locations *ϕ*_*k*_ do not depend on the group *j*. Furthermore, this method allows groups to share statistical strength via the atom weights *v*_*k*_ of the base distribution *G*_0_. Indeed, the vector of weights for each group *j* can be obtained as *π*_*j*_ ∼ DP(*α*, *v*).

In the corresponding culinary metaphor, the HDP can be explained with a Chinese restaurant franchise (CRF), in which there is a collection of restaurants, and dishes are shared across restaurants. However, the popularity of each dish, i.e., the corresponding atom weight, is different in each of the restaurants [6].

### 3.2 Single-p dependent Dirichlet process

The single-p DDP is another DDP, which works in a complementary fashion to the HDP. In this case, atom weights are shared across groups while atom locations are allowed to vary across groups. In terms of the often used culinary metaphor for DPs [2], the HDP shares the dishes across restaurants but allows a different dish popularity in each of the restaurants, while the single-p DDP shares the dish popularity across restaurants but allows the dishes to vary slightly, in order to better fit each group of customers. The latter would be a peculiar CRF in which the popularity of tables is matched one-to-one across restaurants, with the served dish in each linked table slightly customized in each restaurant (e.g., different ingredients, cooking time or local taste).

In the single-p DDP, the latent measure for each group *j* can be expressed as

$$G_{j}=\sum_{k=1}^{\infty }\pi_{k}\delta_{\phi_{jk}},$$ (4)

where the vector *π* ∼ GEM(*α*) contains the mixture weights and *ϕ*_*jk*_ are the atom locations. The single-p DDP does not specify how to tie the atom locations *ϕ*_*jk*_ across groups for each *k*. This step is critical, as it conditions the performance of the model. We put forward in Section 4 another stochastic process for this purpose.

## 4 Modeling of the finishing time

### 4.1 Atom-dependent Dirichlet process mixture model

This section describes a BNP model based on the single-p DDP prior that allows comparing the shape of different distributions while keeping the corresponding quantiles fixed. In the context of the marathon, we use it to obtain a fair comparison between runners regardless of their age or gender. Runners are grouped together according to their age and gender, yielding *J* different groups. In our infinite mixture model, we cluster the runners of all groups with a potentially unbounded number of clusters. Each cluster *k* presents a fixed percentage of runners given by *π*_*k*_, with a stochastic process linking the atom locations, i.e., the mean finishing time. This construction has the potential to provide a direct comparison for the finishing time in each group *j*. However, as the single-p DDP is a very general prior, we need to define the likelihood and the stochastic process in a way that is insightful about the marathoner’s finishing time.

We denote each marathoner finishing time as *x*_*ji*_, where *j* = 1, …, *J* indexes the group and *i* runs over marathoners, and we assume a Gaussian likelihood for the finishing time *x*_*ji*_:

$$\begin{array}{ccc} x_{ji}|c_{ji}=k,\mu_{k},\theta_{j},\sigma_{x}^{2} & ∼ & N\left(x_{ji}|\mu_{k}+\theta_{j},\sigma_{x}^{2}\right). \end{array}$$ (5)

Here, *μ*_*k*_ denotes the global mean for cluster *k*, *θ*_*j*_ is the shift associated to group *j*, *c*_*ji*_ represents the cluster assignment associated to observation *x*_*ji*_, and $\sigma_{x}^{2}$ is the variance of the Gaussian distributions. Hence, we use a cluster-specific parameter *μ*_*k*_ to describe cluster *k*, but we allow deviations from this value due to age or gender (this effect is modeled by *θ*_*j*_).

The key aspect that makes the single-p DDP useful for comparing different age-gender groups is the stochastic process that governs *θ*_*j*_. We would like this value to vary smoothly across ages, and therefore we choose a zero-mean Gaussian process prior for it:

$$\theta ∼N\left(0,\Sigma_{\theta }\right),$$ (6)

where ***θ*** = [*θ*_1_, …, *θ*_*J*_]^⊤^, and for the covariance function we use the standard choice, i.e., the squared exponential kernel given by

$${\left(\Sigma_{\theta }\right)}_{\ell j}=\sigma_{\theta }^{2}\cdot \exp{\left(-\frac{\left(\ell -j\right)}{2\nu^{2}}\right)}^{2}+\kappa \delta \left(\ell -j\right),$$ (7)

where *ℓ* and *j* represent two different age groups, $\sigma_{\theta }^{2}$ accounts for the variance, *ν* controls the degree of correlation between age groups *ℓ* and *j*, and *κ* is a jitter factor to avoid numerical instabilities. We use an independent Gaussian process for each gender. There are other alternatives, see [15] for a comprehensive introduction for valid covariance functions. In our case, the squared exponential kernel is a smooth kernel (infinitely differentiable) that captures the correlation between the different age groups.

Finally, we place the following priors over the assignment variables *c*_*ji*_ and the cluster weights ***π***:

$$c_{ji}|\pi ∼\pi ,$$ (8)

and

π|α∼GEM(α), (9)

**Fig 2:**
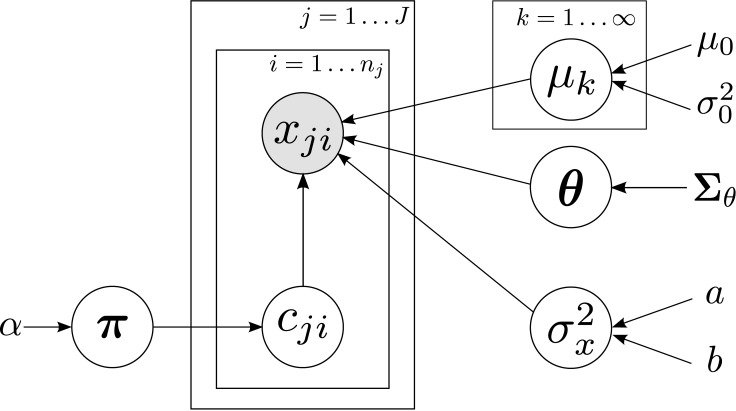
Graphical representation of the basic ADDP mixture model. Grey circles represent observed variables, white circles are hidden random variables. Plates refer to duplicated random variables.

We refer to this single-p DDP prior, together with the likelihood model in Eq (5) and the Gaussian process for *θ*_*j*_, as the atom-dependent Dirichlet process (ADDP) mixture model. This model is similar to the ANOVA-DDP prior in [24]. We rely on a Gaussian process to control the dependency between the shift variables *θ*_*j*_, while the ANOVA-DDP prior relies on independent priors. This allows for smooth variations of the cluster means with the age. A graphical representation for the ADDP is depicted in Fig 2.

We place a Gaussian prior over the cluster means *μ*_*k*_ and an inverse gamma prior over the variance $\sigma_{x}^{2}$, i.e.,

$$\mu_{k}∼N\left(\mu_{0},\sigma_{0}^{2}\right),$$ (10)

$$\sigma_{x}^{2}∼\text{IG}\left(a,b\right),$$ (11)

where *μ*_0_, $\sigma_{0}^{2}$, *a* and *b* are hyperparameters of the model. The value of $\sigma_{0}^{2}$ is assumed to be much larger than $\sigma_{\theta }^{2}$, so that the first one controls the overall finishing time for the clusters (hours), whilst $\sigma_{\theta }^{2}$ controls the differences between groups due to different ages (minutes). We assume a common variance $\sigma_{x}^{2}$ for all the clusters, because it provides an ordering of the clusters, which is necessary for comparing the finishing times. Allowing for different variances for each component should provide a more accurate (in the sense of better density estimation) description of the finishing times, but a less interpretable and less actionable representation, as runners assigned to Gaussian components with different variances are not directly comparable. We have not placed a joint prior for the cluster means and variances through the normal-inverse gamma distribution, since separate priors might have better properties for density estimation [25] and allow for faster Gibbs sampling inference.

### 4.2 Model extensions

#### Multiple races

**Fig 3:**
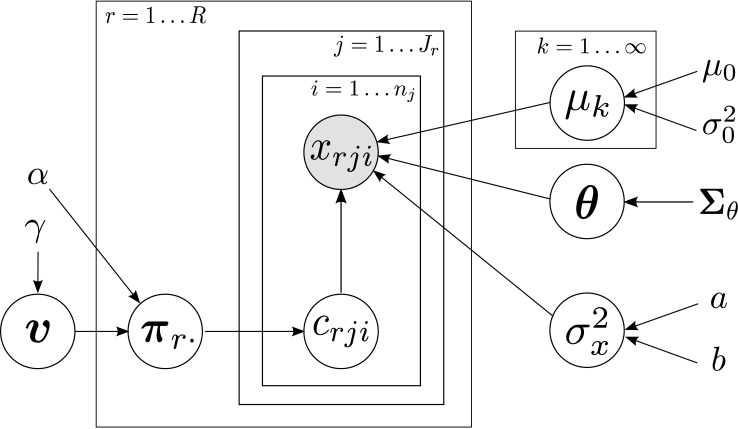
Graphical representation of the H-ADDP mixture model. Cluster weights change across races, whereas cluster means change across age-gender groups.

We could apply the previous models to finishing times from different races or years, but we might obtain unexpected results since different races can present unalike conditions due to temperature, elevation profile, humidity, pull of runners, etc. In this section, we present an useful extension that deals with different races all together and allows drawing comparisons between these races. In order to deal with this, we extend the basic ADDP model using varying weights across races. This leads to a hierarchical ADDP model, in which cluster weights are allowed to change across races, and cluster parameters are allowed to change across age-gender groups. We refer to this model as the hierarchical ADDP (H-ADDP) model. Fig 3 shows a graphical representation of this extended model, with the following likelihood and priors:

$$\begin{array}{ccc} x_{rji}|c_{rji}=k,\mu_{k},\theta_{j},\sigma_{x}^{2} & ∼ & N\left(x_{rji}|\mu_{k}+\theta_{j},\sigma_{x}^{2}\right),\\ \;\\ \mu_{k} & ∼ & N\left(\mu_{0},\sigma_{0}^{2}\right),\\ \theta & ∼ & N\left(0,\Sigma_{\theta }\right),\\ \sigma_{x}^{2} & ∼ & \text{IG}\left(a,b\right),\\ c_{rji}|\pi_{r\cdot } & ∼ & \pi_{r\cdot },\\ \pi_{r\cdot }|v,\alpha & ∼ & \text{DP}(\alpha ,v),\\ v|\gamma & ∼ & \mathrm{GEM}(\gamma ), \end{array}$$ (12)

where *r* indexes the different races, *γ* is the upper level concentration parameter, $\pi_{r\cdot }={\left(\pi_{rk}\right)}_{k=1}^{\infty }$ are the mixture weights for race *r*, $v={\left(v_{k}\right)}_{k=1}^{\infty }$ are the global weights for all races.

One simple way to interpret this model is by conditioning on a particular race or an age-gender group. If we only have data from a single race, we recover our original ADDP model. If we only have data from a single age-gender group, we recover an HDP, i.e., the cluster components are shared, but the mixing proportions are different.

#### Age-gender interaction

The basic ADDP mixture model considers male and female runners independently, assuming independent shift delays *θ*_*j*_ between both genders *j* (i.e., we use a block-diagonal covariance matrix). A natural extension of the model consists in introducing an additional gender factor *δ* and some age-gender interaction factors *ω*_*j*_ in order to capture the correlation between male and female athletes. In such model, the shift delays *θ*_*j*_ are shared for both men and women, and *j* indexes different *age* groups instead of *age-gender* groups. We refer to this model as the age-gender interaction ADDP model. The generative model can be written as follows:

$$\begin{array}{ccc} x_{ji}|c_{ji}=k,g_{ji},\mu_{k},\theta_{j},\sigma_{x}^{2},\delta ,\omega_{j} & ∼ & N\left(x_{ji}|\mu_{k}+\theta_{j}+g_{ji}\left(\delta +\omega_{j}\right),\sigma_{x}^{2}\right),\\ \;\\ \mu_{k} & ∼ & N\left(\mu_{0},\sigma_{0}^{2}\right),\\ \theta & ∼ & N\left(0,\Sigma_{\theta }\right),\\ \sigma_{x}^{2} & ∼ & \text{IG}\left(a,b\right),\\ c_{ji}|\pi & ∼ & \pi ,\\ \pi |\alpha & ∼ & \mathrm{GEM}(\alpha ),\\ \delta & ∼ & N\left(0,\sigma_{g}^{2}\right),\\ \omega \; & ∼ & N\left(0,\Sigma_{\omega }\right), \end{array}$$ (13)

where the indicator variables *g*_*ji*_ differentiate male (*g*_*ji*_ = 0) and female (*g*_*ji*_ = 1) runners, *δ* is the gender effect, and ***ω*** = [*ω*_1_, …, *ω*_*J*_]^⊤^ contains the age-gender interaction factors influencing the likelihood of female runners. Additionally, $\sigma_{g}^{2}$ and **Σ**_*ω*_ are hyperparameters of the model.

#### Cluster-dependent shifts

We now present another extension of the model concerning the shifts ***θ***. In the model described above, the delay *θ*_*j*_ only depends on the age and gender, which implies that the shift is the same for all clusters *k*, no matter whether they are fast or slow runners. However, we can also consider cluster-dependent shifts. This allows us to capture different shift evolutions across age/gender depending on the speed of runners. That is, instead of having a single delay *θ*_*j*_ for each group, we consider a different delay *θ*_*jk*_ for each cluster and group. Each vector ***θ***_⋅*k*_ = [*θ*_1*k*_, …, *θ*_*Jk*_]^⊤^ follows its own Gaussian process with mean ***μ***_*θ*_ and covariance matrix **Σ**_*θ*_:

$$\theta_{\cdot k}∼N\left(\mu_{\theta },\Sigma_{\theta }\right).$$ (14)

Table 1 shows a summary of all the ADDP model proposed in this paper. The main difference between them is the way in which the atom locations vary across the covariate space, i.e., the age-gender space. In the case of the H-ADDP model, atom positions depend on the age and gender of runners, and weights vary as a function of the race.

**Table 1:** Summary of the ADDP mixture models developed in this paper.

Model	Atom weights	Atom locations
Basic ADDP	*π*_*k*_	*μ*_*k*_ + *θ*_*j*_
H-ADDP	*π*_*rk*_	*μ*_*k*_ + *θ*_*j*_
Age-gender interaction ADDP	*π*_*k*_	*μ*_*k*_ + *θ*_*j*_ + *g*_*ji*_(*δ* + *ω*_*j*_)
Cluster-dependent ADDP	*π*_*k*_	*μ*_*k*_ + *θ*_*jk*_

## 5 Modeling of running patterns

In this section, we propose an HDP model as a tool that complements the analysis of the previously introduced ADDP. In particular, we address the analysis of temporal evolution of runners during the race, in order to understand how the marathoners pace themselves to complete the marathon. We aim at discovering running patterns, i.e., distinguishing those overly optimistic runners, with decreasing speed along the race, from well-trained runners who tend to keep a constant speed. It also helps to understand where the marathons are harder, so that runners can know beforehand. The hidden running patterns can be used for training purposes, as they can find out the typical shortcomings of athletes with respect to their age, which may help runners train and run more intelligently. In addition, we also show that discovering running patterns provides a new tool for prediction of finishing times, with results comparable to the best reported method in [17].

The idea is to cluster the data according to the relative time spent in each interval regardless of each runner’s total time. In this sense, we are no longer interested in the absolute times, but in the time proportions invested for each interval. Marathons tend to record the elapsed time every 5 kilometers, in addition to half and full-marathon times. Our input data in this case consists of an *N* × *D* dimensional matrix **X** with the time spent for each interval, together with the age and gender. Here, *N* is the number of runners and *D* denotes the number of available time records.

We normalize our input data so that each runner is represented as a vector containing the fraction of time spent for each intermediate interval. We split the data **X** into *J* groups of runners having the same gender and belonging to the same *age group*. Here, we do not use the actual age of the runners, but split them according to age groups (i.e., age ranks) instead. We use the HDP [6] to cluster the running patterns for the different groups. In the HDP, clusters are allowed to show different probabilities for each group, but the per-cluster parameters are shared across groups. As explained in Section 3, we first draw a global base distribution from a DP as *G*_0_ ∼ DP(*γ*, *H*), where $G_{0}=\sum_{k=1}^{\infty }v_{k}\delta_{\phi_{k}}$, and for each group *j* we draw a distribution from a DP using *G*_0_ as base distribution, i.e., *G*_*j*_ ∼ DP(*α*, *G*_0_). In our model, the likelihood function is a Dirichlet distribution and can be written as

$$\begin{array}{c} x_{ji}|c_{ji}=k,p_{k}∼\text{Dirichlet}\left(\tau p_{k1},\ldots ,\tau p_{kD}\right), \end{array}$$ (15)

where **x**_*ji*_ is the normalized *D*-dimensional vector for runner *i* in group *j*, *c*_*ji*_ represents its cluster assignment, **p**_*k*_ = [*p*_*k*1_, …, *p*_*kD*_] is the vector of patterns representing cluster *k*, and *τ* is the concentration hyperparameter of the model. We place a Dirichlet prior over the per-cluster vectors **p**_*k*_,

$$p_{k}∼\text{Dirichlet}\left(\epsilon \ell_{1},\ldots ,\epsilon \ell_{D}\right),$$ (16)

where *ℓ*_*d*_ is the length of interval *d*, and *ϵ* is its concentration hyperparameter.

## 6 Experiments

In the following experiments, we apply the described models to the New York City marathon [26] for 6 different years, between 2006 and 2011. This database consists of 249,899 runners in total. We additionally compare the NYC marathon data to the marathons of Boston [27] and London [28] for 2010 and 2011, including 117,255 additional runners. Data is included in S1 Marathon Database File. In order to test the resulting models, we set aside a test set with 20% of the participants for each race and age/gender group, ensuring that the age and gender proportions are the same in both train and test sets.

**Inference and parameter estimation.** Posterior inference for all simulations is based on Gibbs sampling. Following Algorithm 8 in [29], we do not integrate out the hidden variables, and we propose 10 new clusters at each iteration. Simulations are run in MatLab, with an approximated running time of 2.3 seconds per iteration for the plain ADDP model using an Intel Core i7-4700MQ. In our results in Section 6.1, we report the values of the hidden variables (means and shift delays) averaged for the last 10,000 iterations after running the sampler for 50,000 iterations. In Section 6.2, we run 10,000 iterations of the sampler and average the results for the last 2,000 iterations. For the per-cluster variables, we carry out the averaging procedure to account for potential label switching. Label switching is common in Bayesian mixture models, and it occurs because the likelihood is invariant to permutation of the cluster labels [30, 31]. Such problem might occur in our case, but it is easy to detect by looking at the traceplot of the cluster-specific parameters, and hence parameters across MCMC samples can easily be matched and averaged. Moreover, in our plots we only report the value of the most populous clusters after filtering out the spurious ones with few runners. These spurious clusters appear as a consequence of the nonparametric nature of the model and the inference procedure, and can also be easily detected by looking at the traceplot of the cluster means. Without more data we cannot actually tell whether there is an actual phenomenon in them or they have locked in a noisy pattern.

**Choice of hyperparameters.** In the case of the basic ADDP mixture model in Section 4, we use: $\sigma_{\theta }^{2}=0.05$, *ν* = 10, *κ* = 10^−6^, *a* = 1, *b* = 1, *μ*_0_ = 5 and $\sigma_{0}^{2}=1$. The value of *μ*_0_ and *σ*_0_ are set so that 2-hour marathoners are within 3 standard deviations and 9-hour marathoners (typical cut-off time) are not unheard of. The ratio between *σ*_*θ*_ and *σ*_0_ is approximately 1 to 4, so the former is of the order of 15 minutes, while the latter is of the order of one hour. Therefore, *σ*_0_ controls the overall finishing time, while *σ*_*θ*_ controls the differences among age groups. The values of *a* and *b* control the variance of each Gaussian component and are set so that values of less than an hour, but not too small, are more likely. *ν* was defined in Eq (7) and controls the correlation between the mean finishing time of runners having same gender and different ages, so that they do not deviate significantly. The value of *κ* measures the error in the recorded time and in this case it prevents numerical instabilities. In the age-gender interaction ADDP model, we choose the same hyperparameters as in the basic ADDP model, and the additional hyperparameters are set as $\sigma_{g}^{2}=0.05$ (which corresponds to the same prior variance as for *θ*_*j*_) and $\Sigma_{\omega }=\frac{1}{2}\Sigma_{\theta }$.

For the HDP model in Section 5, we set *ϵ* = 0.2 km^−1^ and *τ* = 5000, because the differences between the relative time spent at each 5-km interval are typically small. Since *τ* plays the role of an inverse variance, the larger its value, the more clusters we should expect. We have found empirically that values of *τ* ranging in the thousands (between 2500 and 10000) provide results that admit similar interpretations, and they only differ in the granularity of the clusters. Finally, we place a Gamma prior with shape 1 and scale 10 over the concentration parameters *α* and *γ*, and sample their values following [32]. These hyperpriors are chosen in order to avoid the creation of too many spurious clusters. Due to the huge amount of data, results are not very sensitive to hyperparameter values, as long as they are not set to completely misleading and unrealistic values. In our application at hand, we can actually set (and be able to explain) the values of the hyperparameters using all our prior knowledge, as detailed above, which is important in order to incorporate the known information and allow for the expected variances.

### 6.1 Modeling of the finishing time

#### Density estimation

**Fig 4:**
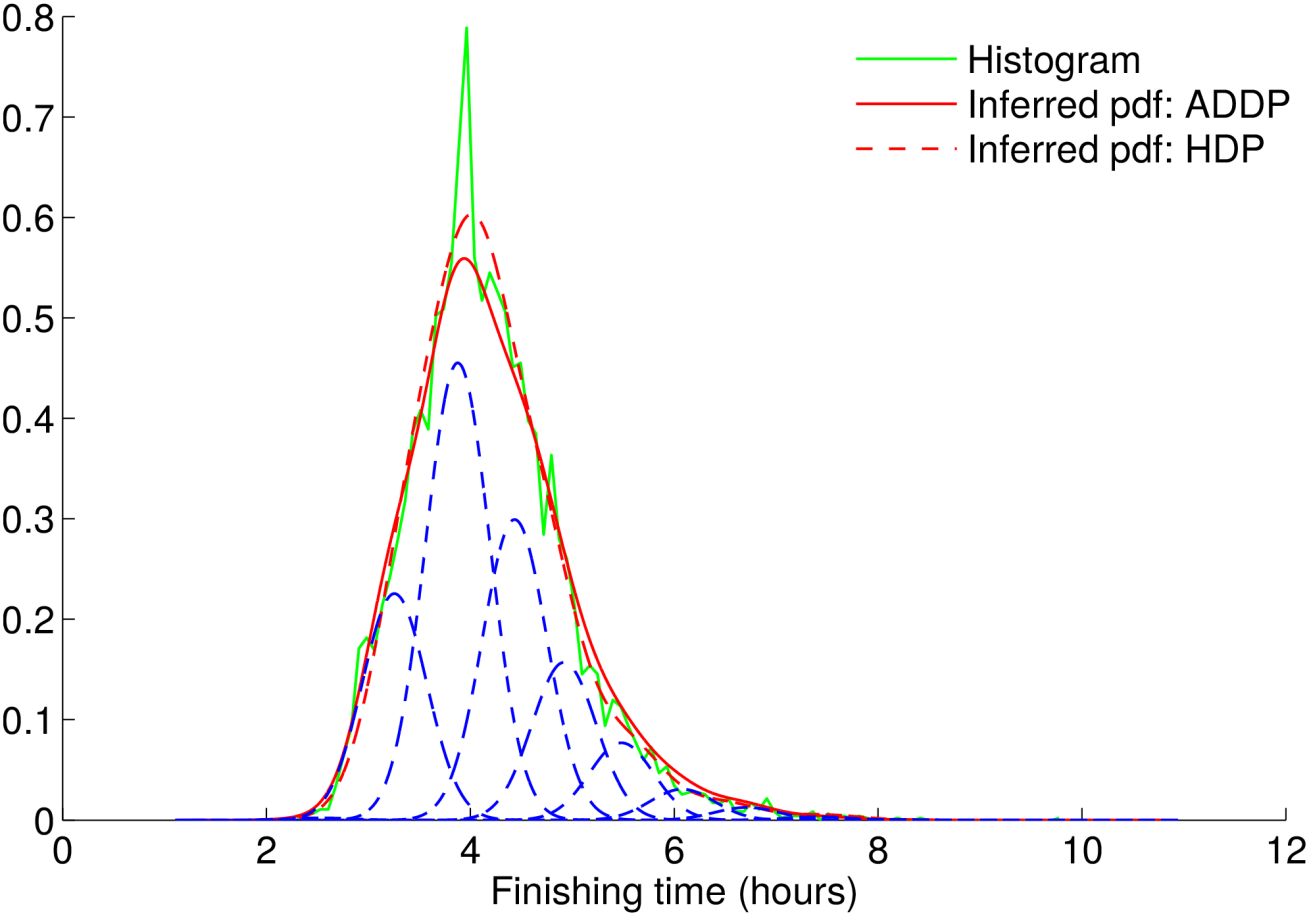
Density estimation capacities for the basic ADDP model. The histogram corresponds to the population of forty-year-old male runners, which is the largest age-gender group. The red curves are the probability density functions inferred by the basic ADDP model. The blue dotted lines represent the inferred individual clusters.

We first show the performance of our basic ADDP mixture model in terms of density estimation, compared to a standard HDP with Gaussian likelihood, see [6] for details. We depict in Fig 4 the empirical histogram for a particular group of runners (we choose all forty year-old male finishers for the plot), together with the overall density estimates, using data for the six considered NYC marathons. We obtain *σ*_*x*_ = 5.8 minutes. Both the ADDP and HDP model perform similarly in terms of density estimation. Note that the empirical histogram in Fig 4 presents one narrow peak that is not fully captured by the ADDP nor HDP model. This peak, just under 4 hours, and the valley right afterwards are due to some runners trying to finish (and succeeding) a sub-4-hour marathon. This is a psychological effect that has limited interest for us, since it is not indicative of runners’ inherent performance. Using cluster-specific values for the variance $\sigma_{x}^{2}$ would yield better density estimation, even capturing this peak, but it would fail to provide any comparison between distributions. Here, we are interested in ordering runners into clusters for comparison, which is achieved by having a shared value of $\sigma_{x}^{2}$ for all clusters.

#### Impact of age and gender

**Fig 5:**
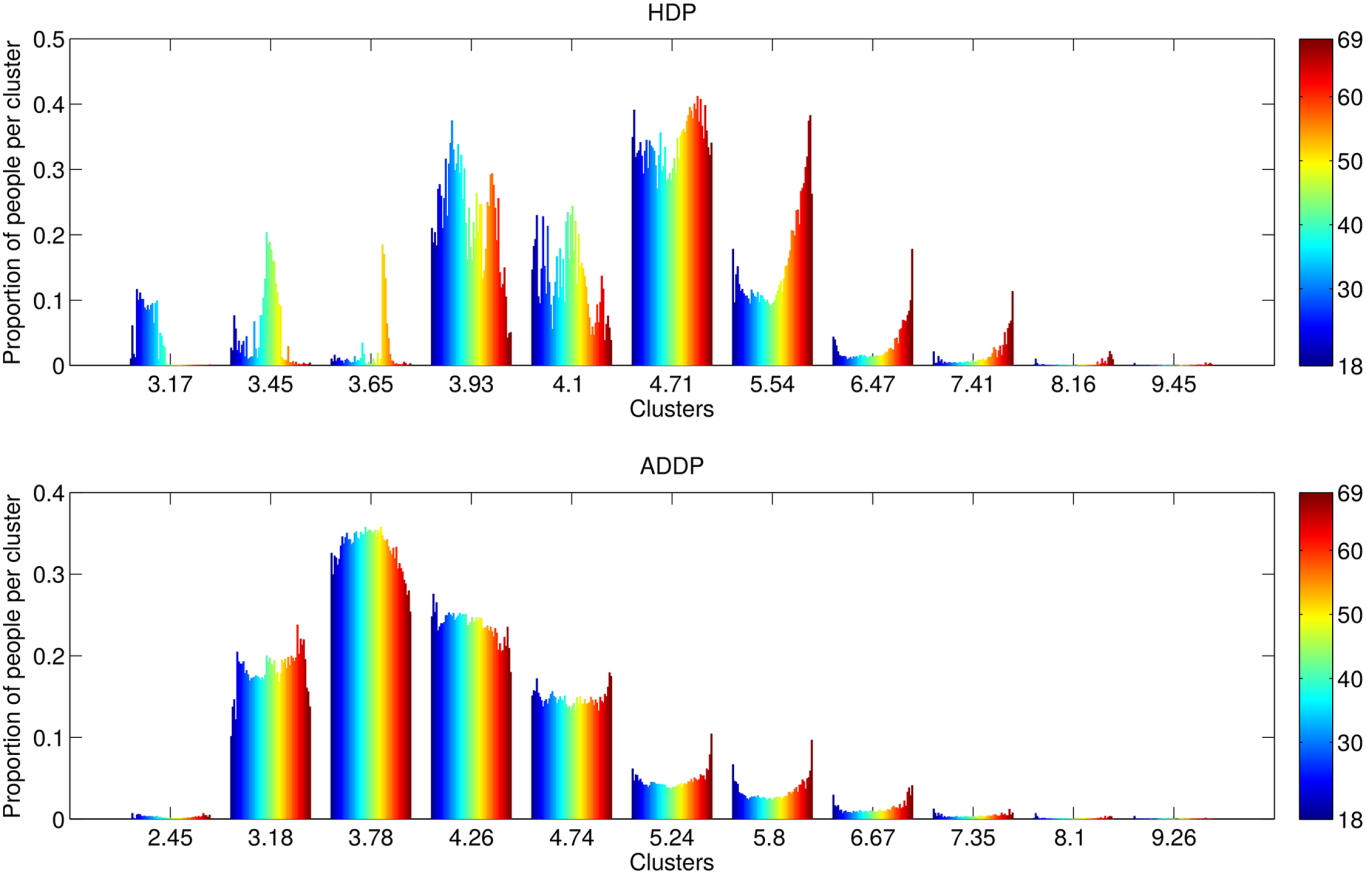
Age proportions per cluster. The HDP is at the top and the age-gender interaction ADDP is at the bottom. Male and female runners are shown together. The colors design the age of the runners, from 18-year-old in blue to 69-year-old in dark red. For the ADDP model, the cluster labels correspond to *μ*_*k*_ + *θ*_*j*_, with the value of *θ*_*j*_ corresponding to the 28-year-old male runners, the shifts for other age or gender can be found in Table 2.

We now use the age-gender interaction ADDP model described in Subsection 4.2. In addition to its density estimation capacity, the ADDP has an additional descriptive strength, since it can show the impact of age and gender on runners performance straightforwardly through inference of the age delays *θ*_*j*_, gender factor *δ* and age-gender interaction factors *ω*_*j*_. Fig 5 shows the average proportion of runners in each cluster, as well as the inferred cluster means *μ*_*k*_, for both the ADDP and a standard HDP with Gaussian likelihood [6]. Runners aged above 69 are not reported because there are too few of them. The HDP results are not easy to interpret, except for the first three clusters that show the time degradation with age, because we do not know the cumulative percentage as the finishing time is increased.

The ADDP model is much easier to understand. The first cluster contains the “Olympic” quality runners for all ages, if Olympics were held for each age group (less than 1% of the runners). The second cluster has the competitive runners (about 13% of the runners), the third cluster has the standard marathoners (about 33% of all runners), and so on. The x-axis in Fig 5 provides the value of *μ*_*k*_ + *θ*_*j*_ for 28 year-old male runners and the degradation for other ages and sex is shown in Table 2. Using the plot and the table, we can know the proportion of runners in each group and how much extra time they need compare to the fastest group.

**Table 2:** Averaged values of *θ*_*j*_ for men (or *θ*_*j*_ + *δ* + *ω*_*j*_ for women) for all age groups in minutes. The values have been shifted so that *θ*_28_ = 0 for men. This table is useful to directly compare the finishing time of two runners of any age and gender. We only have to calculate the difference of their respective cells in the table and we will get the time penalty that should be considered (in minutes).

age	18	19								
men	8.00	6.34								
women	33.60	31.89								
age	20	21	22	23	24	25	26	27	28	29
men	4.88	3.62	2.53	1.66	0.98	0.49	0.18	0.04	0.00	0.10
women	30.43	29.21	28.19	27.42	26.87	26.53	26.38	26.41	26.53	26.79
age	30	31	32	33	34	35	36	37	38	39
men	0.28	0.51	0.79	1.09	1.39	1.71	2.03	2.34	2.65	2.98
women	27.13	27.50	27.87	28.27	28.65	29.03	29.39	29.74	30.08	30.46
age	40	41	42	43	44	45	46	47	48	49
men	3.30	3.66	4.11	4.61	5.25	5.99	6.85	7.90	9.09	10.47
women	30.82	31.23	31.78	32.41	33.21	34.13	35.24	36.58	38.07	39.81
age	50	51	52	53	54	55	56	57	58	59
men	12.01	13.74	15.66	17.74	20.00	22.38	24.91	27.51	30.21	32.95
women	41.73	43.88	46.23	48.75	51.47	54.29	57.26	60.27	63.36	66.44
age	60	61	62	63	64	65	66	67	68	69
men	35.70	38.44	41.16	43.80	46.35	48.77	51.06	53.17	55.09	56.81
women	69.50	72.51	75.46	78.26	80.93	83.42	85.73	87.83	89.69	91.31

**Fig 6:**
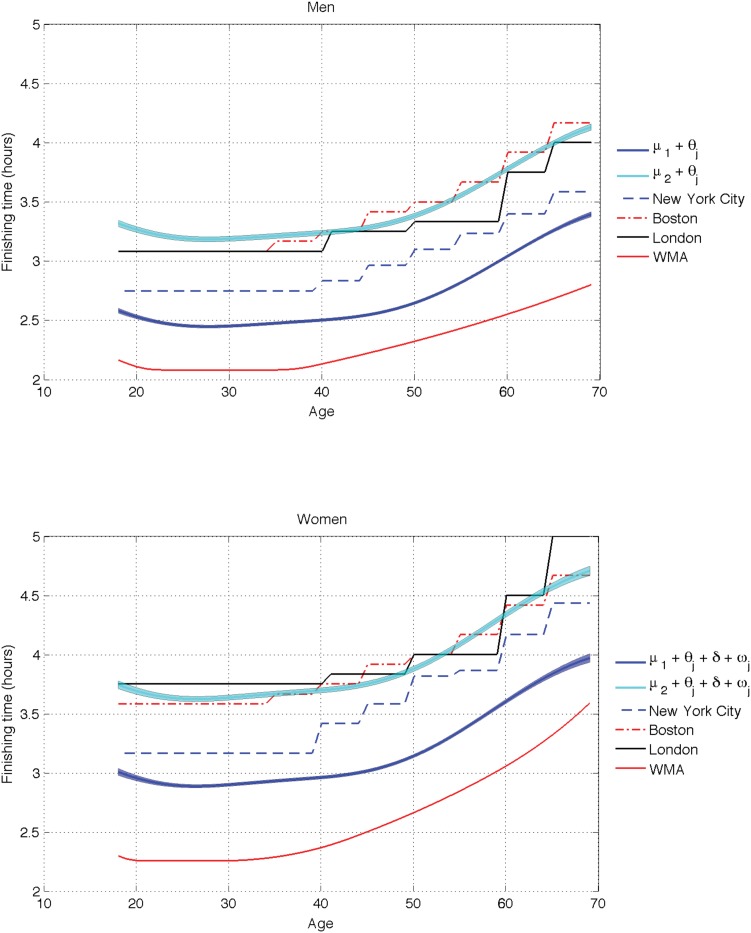
Inferred cluster means and entry requirements. Comparison of the inferred cluster means, i.e., *μ*_*k*_ + *θ*_*j*_ with *k* ∈ {1,2}, with the entry requirements (runners below the curve can qualify) for New York City, Boston and London marathons. (Top) Men. (Bottom) Women.

Fig 6 shows the value of the inferred cluster means *μ*_*k*_ plus and minus one standard deviation, shifted according to *θ*_*j*_, *δ*, and *ω*_*j*_ for both men and women. We only depict the two fastest clusters, and compare the corresponding values of the finishing time with the entry requirements of different marathons and the WMA records. Best performance for females and males is predicted, respectively, at 26 and 28 years old, which is consistent with [33]. The plateau afterwards illustrates a stable period of performance between the 30’s and 40’s for both genders.

All plots behave in a similar way. This is what we meant in the introduction by that *the first insights should not be foreign to us*, so experts in marathon modeling can take other conclusions as plausible. Now, we focus on what is different. The most striking difference is how the entry levels penalize younger male runners, specially runners under 25. To be fairer to the youngest runners, their entry time should be raised (in about 7 minutes compared to the 30 years-old). The Boston marathon entry level is perfectly aligned with our second cluster for 40+ years old men and almost perfectly match the female second cluster, except for the runners under 23. There is a penalty of about 4–7 minutes for runners aged 25–39 and between 7–14 minutes for 18–24 year-old runners. The entry times slightly favor the 45–50 year-old runners.

The London marathon also penalizes excessively runners in their fifties compared to those in their forties and sixties, which seems odd. It is also clear that over 50 (or even 45), the degradation of the finishing times per year is significant enough to merit a finer scale to guarantee entry times (this may also apply for 18–23 years-old). For example, a runner of 60 years old is doing almost 15 minutes less than a runner of 64 (which is a very long time in any marathon). Finally, the WMA curve for men penalizes the older male runners, while for older female runners it seems to have a similar trend than the first cluster of the ADDP model. For younger runners, the difference between the typical women in the olympic cluster and the WMA is larger than the difference between the typical men in the olympic cluster and the WMA.

**Fig 7:**
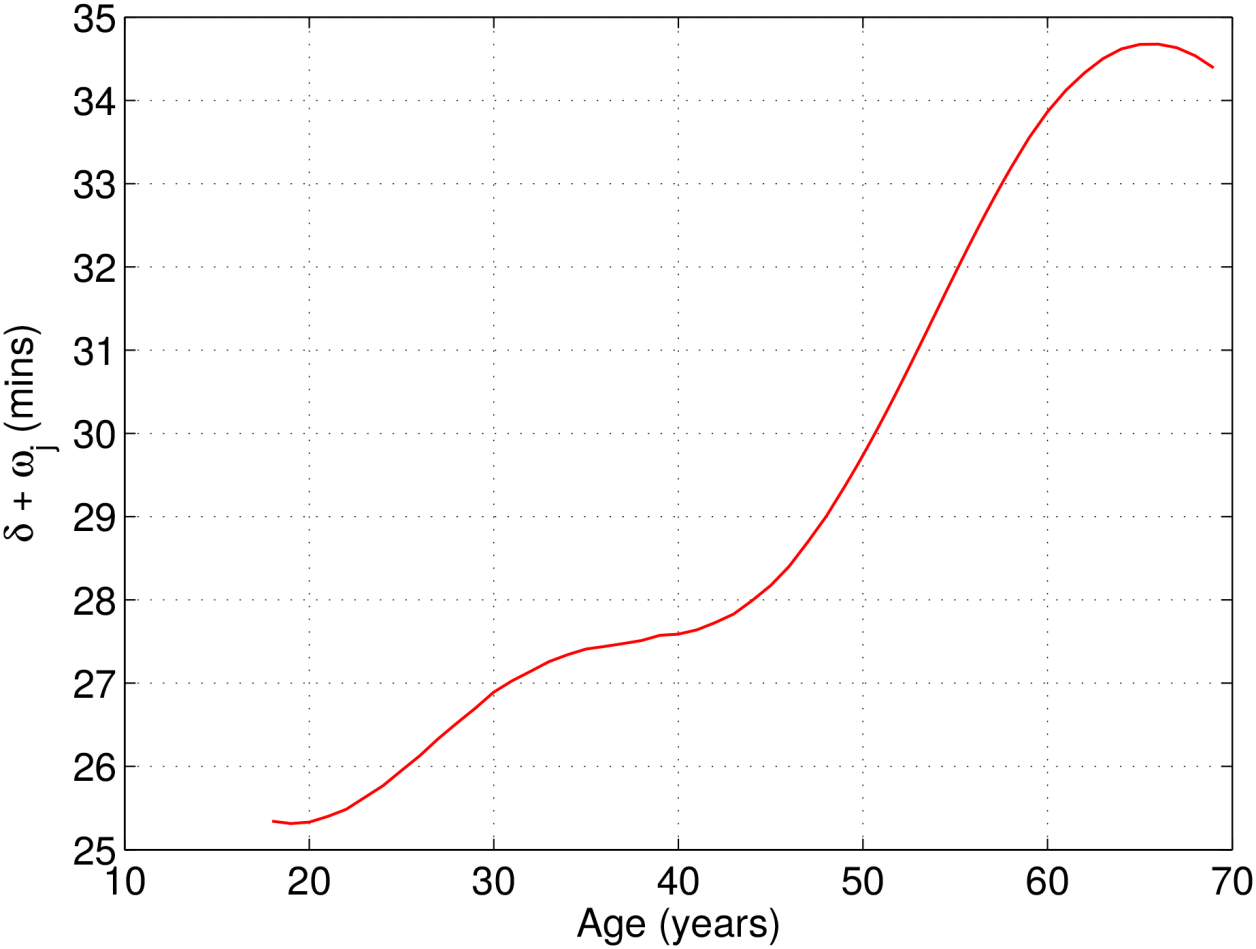
Gender effect on the final performance. Averaged inferred value for the gender coefficients *δ* + *ω*_*j*_.

Fig 7 shows the finishing time gap between women and men. The gap seems to be of about 30 minutes and slightly increasing with age. There are too few runners over 65 for the final decay to be statistically significant. We can come up with two different plausible explanations, but we do not have data to confirm whether this empirical effect is due to any of them or to some other unknown factor. The degradation with age between women and men might be due to physiological factors, i.e., women age differently than men for long distance running, or it might be due to socio-economical factors, i.e., women above 30 cannot train as much as men do.

#### Comparison across multiple races

**Fig 8:**
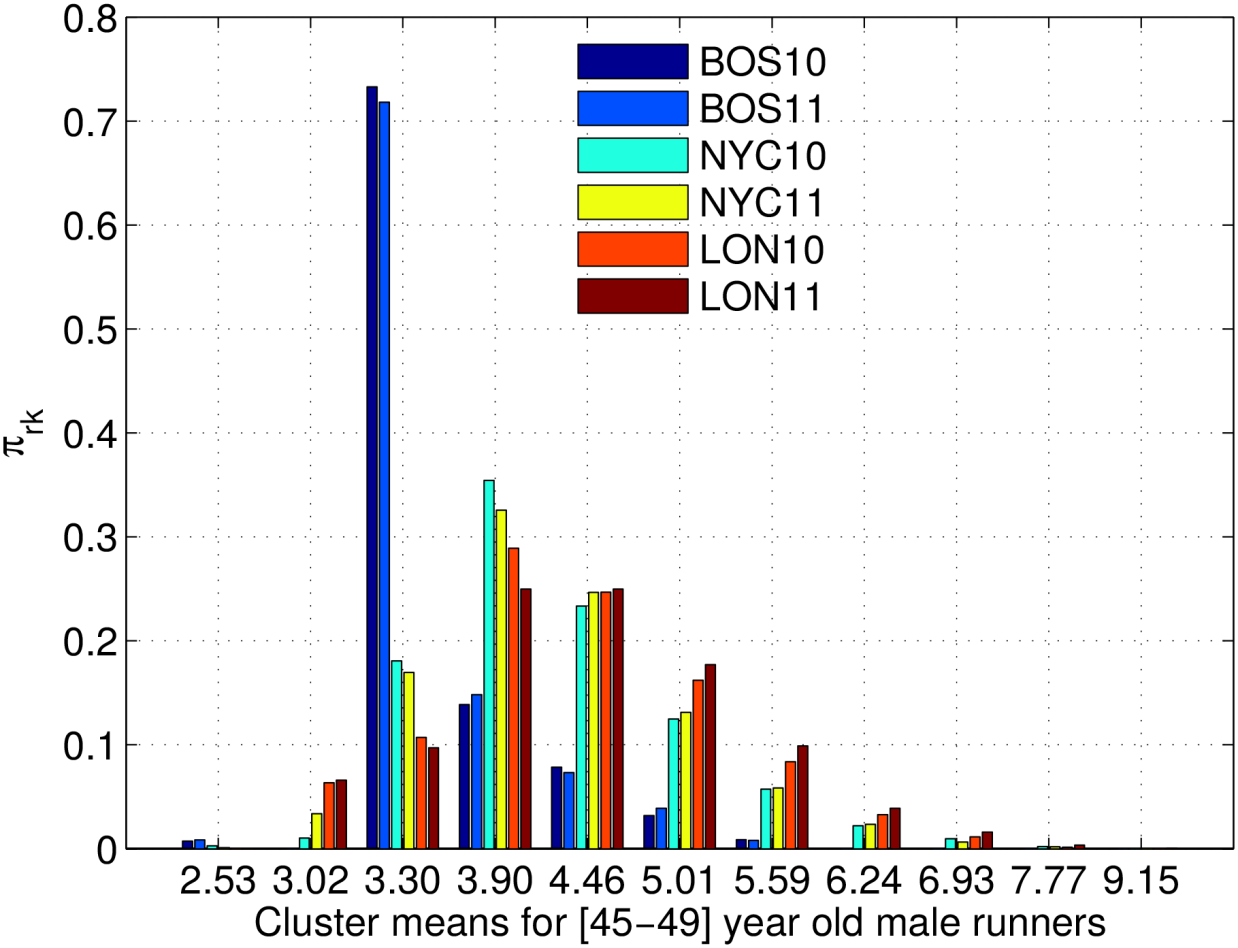
Mixture weights for the H-ADDP mixture model. The figure shows the mixture weights *π*_*rk*_ for each race *r* and cluster *k*. The legend shows the different races, and the x-axis corresponds to different clusters.

We compare the NYC marathons to the ones in Boston and London, using the H-ADDP model described in Section 4.2. We consider both the 2010 and 2011 marathons, and we split the runners into *age groups* instead of using their actual age because we do not have this data available for London marathon. Fig 8 shows the inferred values of the per-race weights *π*_*rk*_. The values for *μ*_*k*_+*θ*_*j*_ in the x-axis are those of the 45–49 male runners and the value of *σ*_*x*_ = 19 minutes. First, we notice that the values of *π*_*rk*_ are quite different for each place, but they show little variation between different years. We can argue that this pattern is mainly due to the race difficulty, assuming a stationary selection of the runners.

Boston has the most striking pattern, which can easily be explained by the strict entry requirement time. For 45–49 years old the entry time is 3h25m and the cluster with 70% of the runners has a mean of 3h19m20s. There is a group of almost 15% of the runners that finish just under 4 hours and about 15% of the runners that do much worse than their qualifying time. This might be due to poor training or having some issue during the race. For the Boston marathons, there are no runners in the 3h cluster and around 1% runners in the fastest cluster. The void in the 3h group is due to the massive proportion of runners in the 3h19m group, which makes any runner in that group to be represented by the 3h19m cluster. The runners under 3h are the runners that do much better than the needed qualifying time and cannot be represented by the massive 3h19m group.

The proportions in NYC and London are more similar to each other, as both marathons allow runners to enter the race in more ways than just by entry requirement. Being these two races more accessible or *democratic*, we can consider the proportions in the different clusters closer to the general population of marathon runners. The 3h group is more populous in London than NYC, but the 3h19m and 3h53m clusters contain a larger proportion of NYC runners. The 4h30m group is equally probable in both races. London seems to attract a higher proportion of slower runners (over 5 hours). This difference might be due to the difficulty of the marathons (profile and weather conditions) or the pull of runners. NYC race is more hilly than London, which can explain the difference in the first cluster, but the runners in NYC are more diverse (coming from different parts of the country and world), while London attracts more local runners. This might also explain the pattern of the slowest athletes.

#### Comparison taking into account the speed

**Fig 9:**
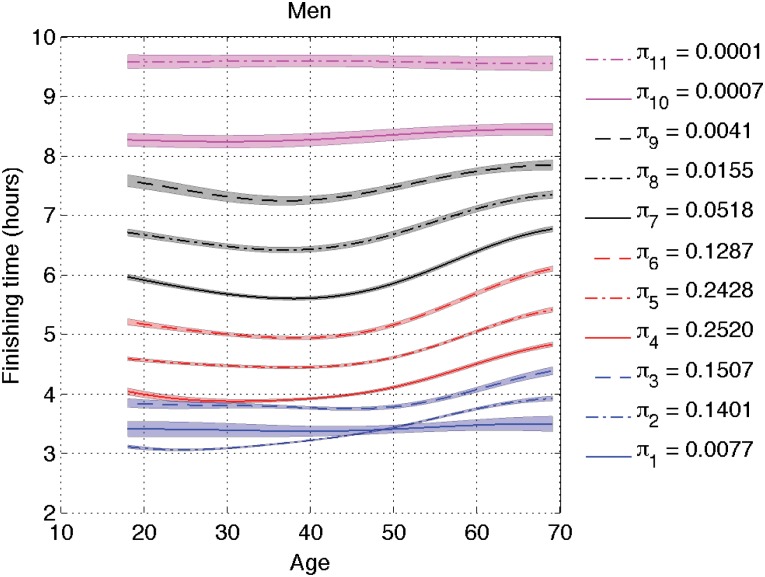
Inferred cluster means *μ*_*k*_ + *θ*_*jk*_ for men. We have used the extended model with speed-dependent clusters. The legend shows the inferred value of the proportions *π*_*k*_ for each cluster.

**Fig 10:**
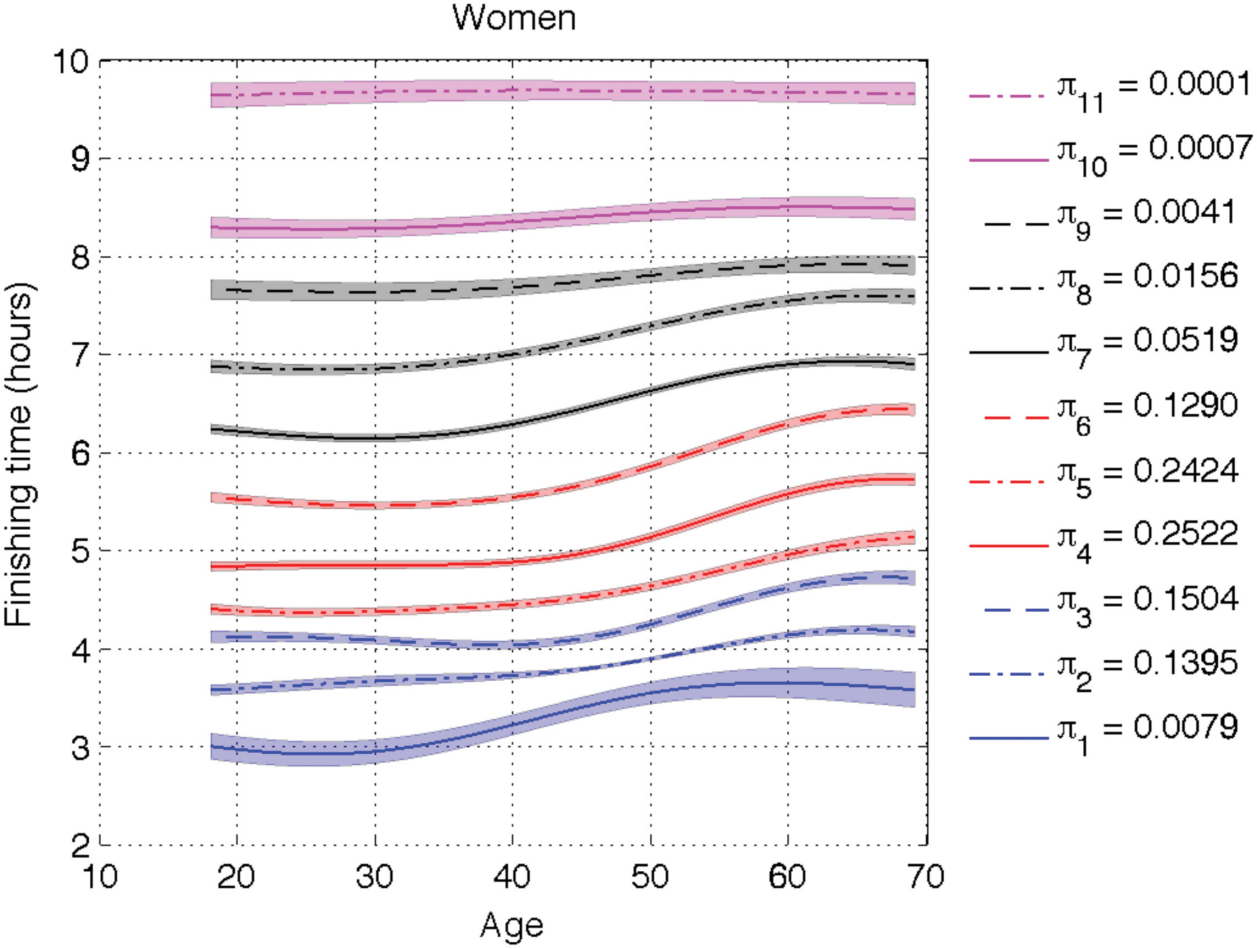
Inferred cluster means *μ*_*k*_ + *θ*_*jk*_ for women. We have used the extended model with speed-dependent clusters. The legend shows the inferred value of the proportions *π*_*k*_ for each cluster.

We now apply the model extension in Section 4.2 with cluster-dependent shift delays *θ*_*jk*_. Figs 9 and 10 show the inferred cluster means for men and women respectively. Although the overall shape of the curves is quite similar across clusters, which validates our previous conclusions, there are some noticeable differences for the fastest runners and we concentrate on those.

The most interesting difference is the behavior of Clusters 1 and 2. At a first glance, it seems to the naked eye that women are faster than men. Cluster 1 captures the Olympic female runners that are doing under 2h45m, and its proportion is very low (less than 1%). Cluster 2 for women covers those runners doing under 3h45m and it represents 13% of the female population. There is a significant difference between Olympic female runners and competitive female runners, so two clusters are needed. For men under 50, Cluster 2 represents 13% of the runners and it captures those doing under 3h30. The model considers that the Olympic runners can be modeled by the tail of the distribution of competitive runners, i.e., Cluster 2, without requiring a new cluster as the Olympic women need. Male runners over 50 behave as women do, and two clusters are needed to separate the Olympic and the competitive groups.

**Fig 11:**
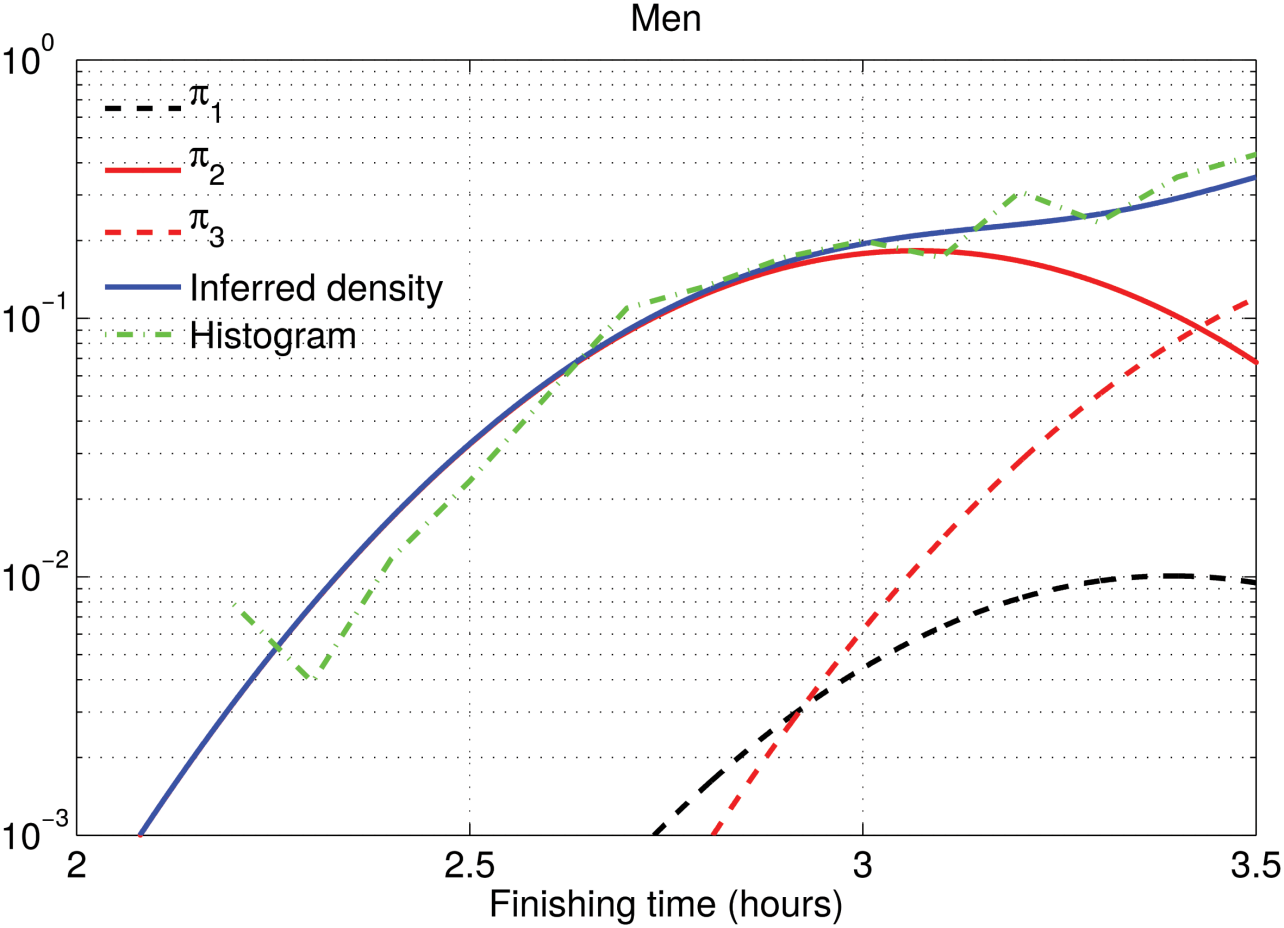
Density estimation for 28-year-old male runners. We have used the cluster-dependent ADDP model, where cluster positions are speed-dependent. The blue solid line represents the inferred distribution and the green dash-dotted line is the normalized histogram with 6-minute bins. Black and red lines correspond to the individual Gaussian components that define the inferred density, weighted by their averaged weight.

**Fig 12:**
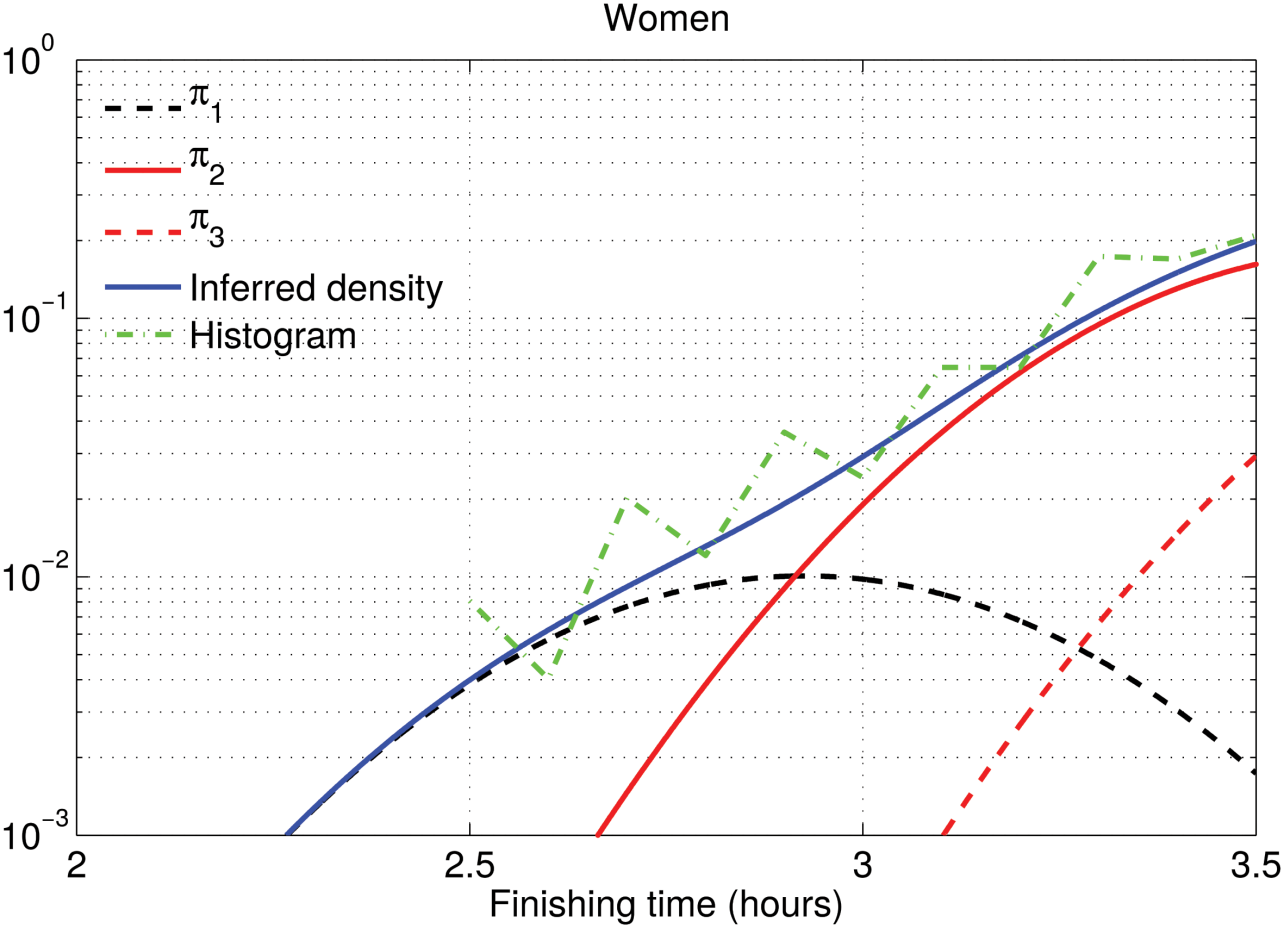
Density estimation for 28-year-old female runners. We have used the cluster-dependent ADDP model, where cluster positions are speed-dependent. The blue solid line represents the inferred distribution and the green dash-dotted line is the normalized histogram with 6-minute bins. Black and red lines correspond to the individual Gaussian components that define the inferred density, weighted by their averaged weight.

For men under 50, Cluster 1 sits in between the two populous clusters, becoming irrelevant in terms of density estimation. It appears because the model forces the same proportion for all clusters across age and gender groups. In order to support this conclusion we have depicted the histogram of the 28-year-old runners together with the inferred density in logarithm scale in Figs 11 and 12. In those plots, we can see that the Olympic male runners just doing over 2 hours can be modeled by the same cluster as the competitive male runners, while the finishing time for the Olympic female runners could not be explained by the competitive female ones, and hence a specific cluster is needed. In previous sections, *θ*_*j*_ was not allowed to vary with *k*. The cluster for the Olympic males was then visible, but this is an effect of forcing the same value of *θ*_*j*_ for all clusters, since women and older male runners need such cluster. This is the only significant difference when we replace *θ*_*j*_ with *θ*_*jk*_. In short, Cluster 1 (with low weight) is describing a different local feature for men under 50 than for men over 50 and women of all ages, and the Olympic male runners below 50 are found in the tail of Cluster 2 instead of Cluster 1 as in the other age/gender groups.

There may be several explanations for this effect. Here we present some hypothesis that should be confirmed with further studies. First, in the NYC marathon the Olympic women run by themselves in an early wave, while the Olympic men start at the same time as everyone else, so competitive men can try to follow them. However, this does not explain the need for a cluster for fast male runners over 50. Second, we can also hypothesize that female Olympic runners have a training that is significantly different from competitive female runners, while for male runners there is a continuum in the training between Olympic and competitive runners. This could also apply for male over 50, in which there are not that many doing Olympic finishing times and competitive runners are not as strong. Finally, another plausible hypothesis is that younger male runners are more risky than female and older male runners. Those that succeed do a better time and close the gap between the Olympic and competitive runners. Older males and female competitive runners do not follow such a risky approach and therefore they do not close the gap with the Olympic runners. There is some evidence on this risky hypothesis in Section 6.2, in which we see that the clusters with reckless running patterns are mainly populated by younger males.

### 6.2 Modeling latent running patterns

Here, we consider temporal sequences of time measurements every 5 km, and at half and full marathon, as explained in Section 5.

#### Running patterns

**Fig 13:**
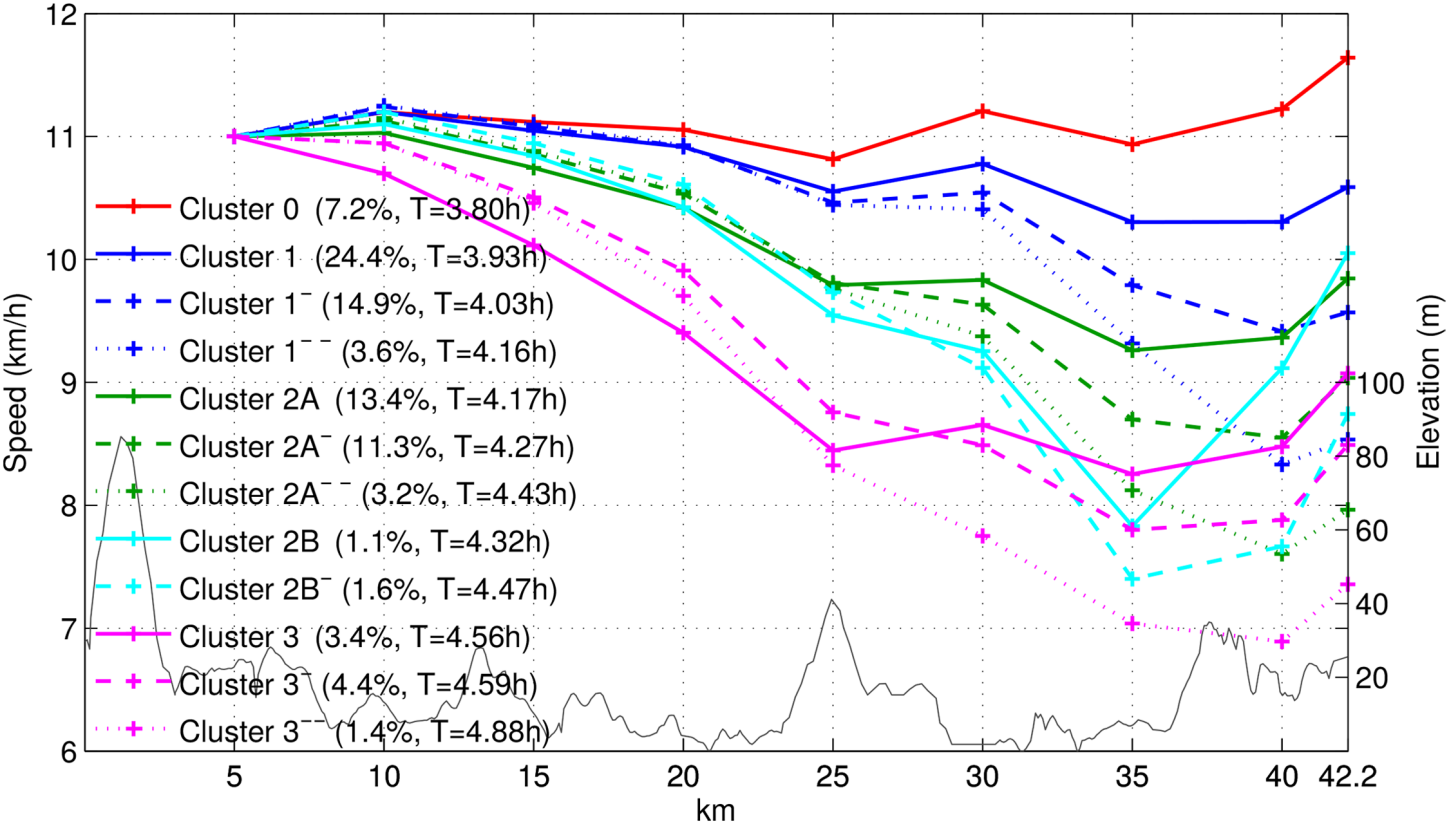
Inferred running patterns. (Thick lines) Inferred running patterns or speed for the twelve most populated clusters, assuming an initial speed of 11 km/h. The legend additionally shows the average proportion of runners in each cluster, as well as the net time for that value of the initial speed. (Thin grey line) Elevation profile of the race.

In this section, we use the data from 2007–2011 NYC marathons, with 194,778 runners. We discarded data from the 2006 marathon because we observed that intermediate measurements were not fully synchronized with the half and full marathon times. After applying our HDP model, we found a modal value of 46 clusters. In Fig 13 we show the twelve most populated clusters, which account for around 90% of the population on average. The remaining clusters do not behave significantly different than the ones we show in this section. For clarity, we do not directly plot the time proportion spent at each interval, but instead show the speed at each 5-km leg, assuming a value of 11 km/h for the speed during the first 5 kilometers. We have removed the half-marathon record for clarity in the plot. The total net time, assuming this value for the initial speed, is shown in the legend for each cluster. We also plot the approximate elevation profile of the marathon with a thin grey line.

Before the half marathon mark, we can roughly see three different types of clusters: those corresponding to athletes running at approximately constant speed (clusters 0, 1, 1^−^ and 1^−−^), those that are already showing a decreased pace (clusters 2A, 2A^−^, 2A^−−^, 2B, and 2B^−^), and those for which the decreased pace is significantly more relevant (clusters 3, 3^−^, and 3^−−^). Just before the 25-km mark, there is an overall drop in performance that can be explained by the Queensborough bridge and, after that, the twelve clusters become clearly different from one another, giving their labels an obvious meaning.

People in Cluster 1 (the most populated cluster, one in every four runner) are well trained runners that run at almost constant speed and the changes can be explained by the hills in each 5-Km interval and they speed up to finish a strong race in the last kilometer, while Clusters 1^−^ and 1^−−^ suffered the effect of the Manhattan hills and bridges in and out of the Bronx, besides the natural weariness after running for 35 km. Cluster 1^−−^ correspond to the runners that outpaced themselves and finished the marathon at a very low speed, compared with what they could have done. Cluster 0 corresponds to runners who could have done a better race if they had run faster from the beginning and not only after half the race. The rest of the clusters correspond to those overly optimistic runners who could not run as fast as they thought at the beginning. These are the runners who suffered the most. For all the clusters, we can observe an increased speed in the last 2 km, which can be explained by the proximity to the finishing line and the effect of trying to finish under some target time.

**Fig 14:**
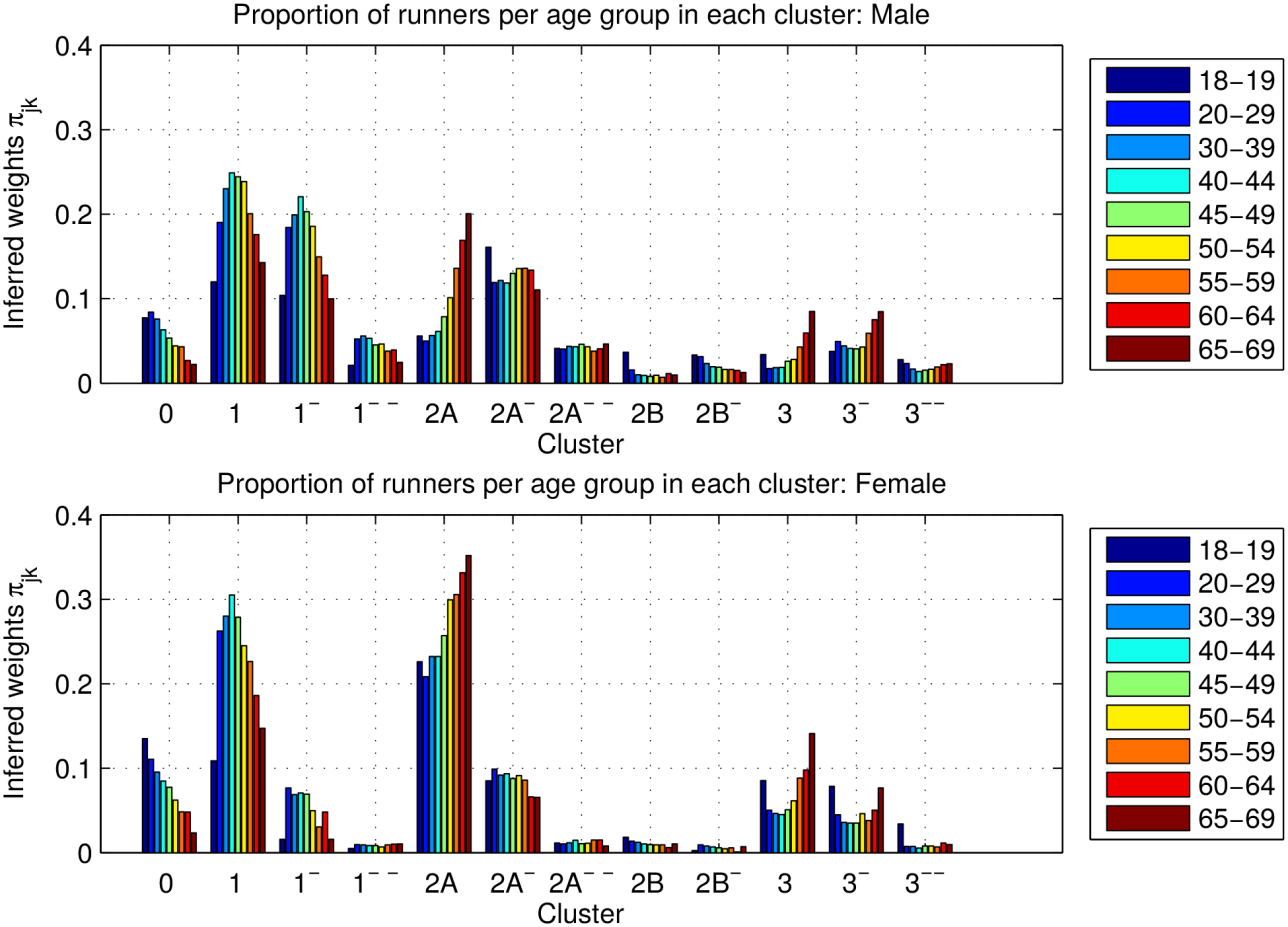
Age proportions for each running pattern. (Top) Men. (Bottom) Women. The legend shows the different age groups considered.

Fig 14 shows the averaged inferred proportion of runners *π*_*jk*_ in each of the twelve most populated clusters for both men and women, broken down by age groups (blue represents the youngest runners). Clusters 1^−^, 1^−−^, 2A^−^, 2A^−−^ and 2B^−^ are mostly populated by men (e.g., 19.4% of men and only 6.7% of women are in Cluster 1^−^, and 5% of men and 0.9% of women are in cluster 1^−−^). In other words, the clusters of overconfident runners are mostly populated by men. Clusters 2A^−^ and 2A^−−^ present a constant proportion across ages for both genders. The proportion of women in Clusters 0 and 2A is higher than for men (e.g., 7.5% of men and 24.1% of women are in Cluster 2A). These clusters represent the conservative runners that have some doubts about how fast they can finish a 42.2-km race. In Cluster 0 there is a larger proportion of 18–19 year-old runners for both genders. These are probably first timers, which is consistent with the inexperienced behavior of runners in that cluster. In contrast, Cluster 1 (well-trained athletes) is mostly populated by runners in their thirties, forties and fifties. Custer 2A becomes more popular for older runners. In this cluster, the runner speed slightly decreases in the first part of the race, but it remains constant in the second half-marathon, which might indicate that after the initial 10 km, the runners slow down to make sure that they can continue at a somewhat constant pace.

**Fig 15:**
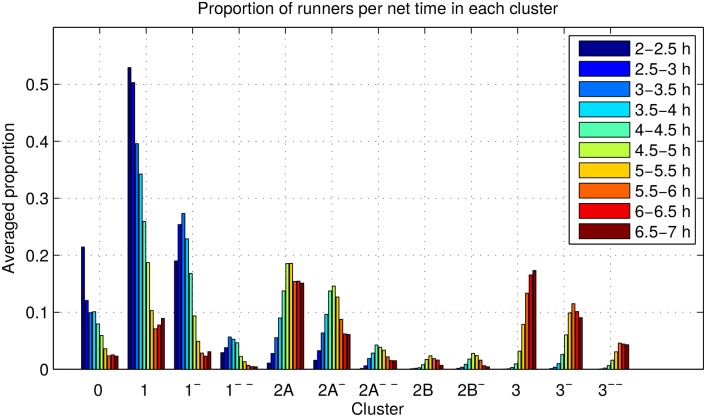
Proportion of runners for each running pattern broken down by net time. The x-axis indexes the clusters found by the HDP model. The legend shows time intervals for the marathon finishing time.

In Fig 15, we show the averaged proportion of runners in each of the twelve most populated clusters, broken down by their net time, up to 7 hours. As expected, Clusters 0, 1 and 1^−^ comprise a high proportion of the fastest runners, i.e., those that can complete the marathon below 4 hours. In contrast, Clusters 3, 3^−^ and 3^−−^ are mostly populated by the slowest runners, with a net time above 5.5 hours. Clusters 2A, 2A^−^, 2A^−−^, 2B and 2B^−^ have the highest proportion of runners with net time between 4.5 and 5.5 hours. These results are consistent with the description of the clusters provided above.

#### Prediction of final performance

We can also apply our model to predict the arrival time of athletes. In this case, observations correspond to time proportions at each interval, up to the last available record. We train our model with the subjects in both the test and the training set, assuming that observations up to interval *D* are known for all of them. Note that we apply our inference procedure independently for each value of *D*. Regarding the prediction task, we apply a Bayesian approach in which we take into account the weights from the posterior probabilities of being in each cluster. At each iteration of the sampler and for each runner in the test set, we first compute the posterior probability of being in each of the clusters found using the training set. Second, we project forward his last available time record to obtain the predicted finishing time for each cluster. Third, our prediction is computed as the weighted average of the predictions for each cluster (weighted by the posterior probabilities of belonging to each cluster). In order to project forward the last available time record for each cluster, we multiply by a factor the time up to interval *D* of the considered runner, i.e., our prediction is obtained as prediction_*ji*_ = *q* × (**x**_*ji*_)_*D*_. The factor *q* is computed as the median of the quotient of the finishing time and the time up to interval *D* for those runners in the training set and in the corresponding cluster. Using the median instead of the mean makes predictions more robust against outlier runners. Finally, we average our predictions for the last iterations of the sampler.

**Fig 16:**
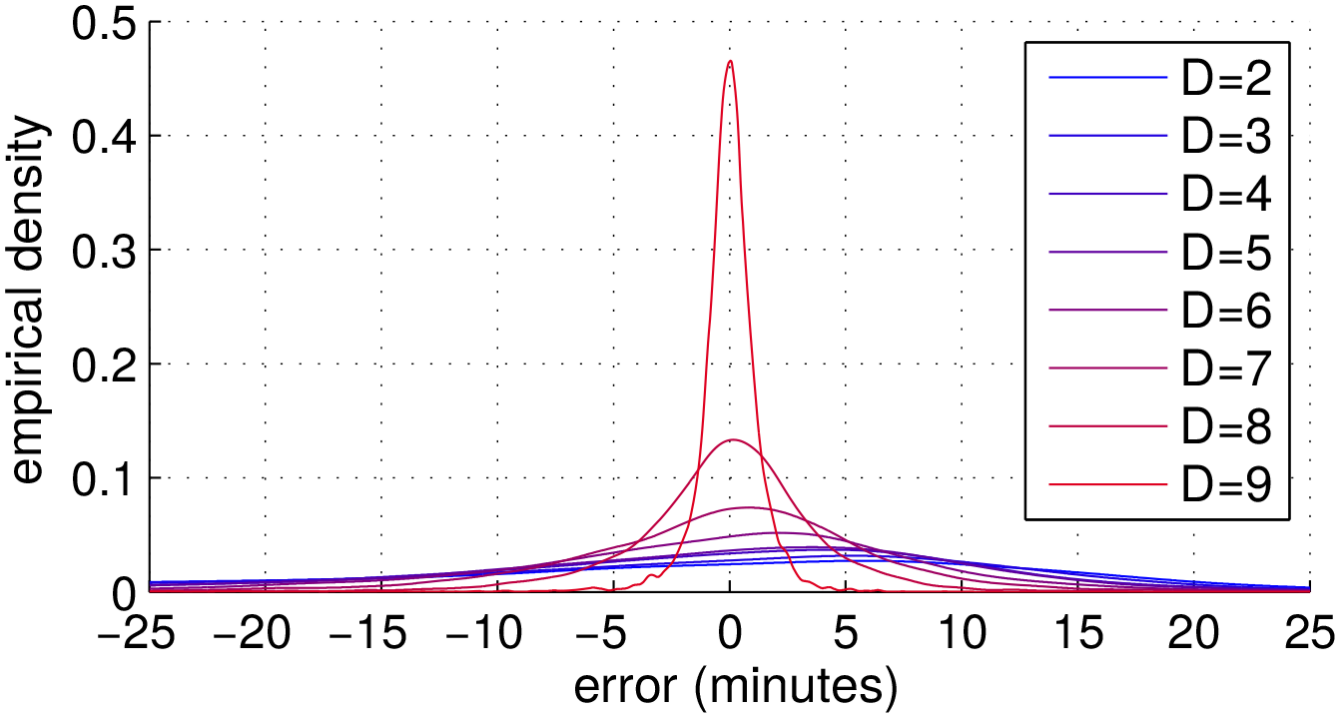
Prediction error. Density of the prediction error for different values of *D* (number of available intermediate records).

In Fig 16, we show the empirical density of the prediction error for all subjects in the test set of the 2011 NYC marathon. As the number of available records *D* increases, the curves tend to shrink around zero.

Tables 3 and 4 report the average prediction errors, as well as the root of the mean square error, compared with the results obtained following the 100-NN method with forward projection described in [17], for 2010 and 2011 NYC marathons. We do not outperform the discriminative method, but our proposal has the advantage of dealing with time proportions instead of absolute times, which allows predictions for slower runners based on the arrival time of faster ones. Although our model only uses relative times (it has one less degree of freedom) it does equally well, the differences being negligible. Both methods are basically unbiased, as the bias only explains less than 2% of the root of the mean square error, but this bias seems to be always positive, which means that the estimations are optimistic on average.

**Table 3:** Test prediction errors for year 2010.

	100-NN		HDP	
	avg	rmse	avg	rmse
*D* = 1	2.819	19.033	3.778	19.533
*D* = 2	2.554	16.586	3.226	17.224
*D* = 3	1.842	13.408	2.207	14.290
*D* = 4	1.717	12.620	2.000	13.436
*D* = 5	1.264	9.748	1.255	10.536
*D* = 6	0.733	6.913	0.705	7.448
*D* = 7	0.221	3.921	0.212	4.195
*D* = 8	0.031	1.355	0.037	1.434

We show the average error for both 100-NN and HDP methods (“avg”), as well as the square root of the mean square error (“rmse”). Results are all expressed in minutes. Rows represent number of available time records.

**Table 4:** Test prediction errors for year 2011.

	100-NN		HDP	
	avg	rmse	avg	rmse
*D* = 1	3.384	20.419	4.247	20.104
*D* = 2	2.777	17.124	3.368	17.610
*D* = 3	2.121	13.833	2.428	14.746
*D* = 4	1.942	13.019	2.219	13.788
*D* = 5	1.624	10.202	1.625	10.962
*D* = 6	0.863	7.279	0.821	7.837
*D* = 7	0.283	4.117	0.269	4.440
*D* = 8	0.035	1.394	0.046	1.471

We show the average error for both 100-NN and HDP methods (“avg”), as well as the square root of the mean square error (“rmse”). Results are all expressed in minutes. Rows represent number of available time records.

## 7 Conclusions

This paper presents a novel application of BNPs to model marathon runners. By including constraints over two well-known BNP priors, we have provided insightful solutions to the problems of age-grading and performance estimation in marathon races.

For the age-grading problem, the ADDP model informs us of the impact of age and gender on runners’ performance. Statistical age-grading curves have been inferred, which allows us to rank and reward athletes fairly in official events. A model extension lets us capture the impact of meteorological conditions or even topology on runners. We infer the latent difficulty for each competition at different years, allowing robust comparison across different marathon events.

We have also applied the HDP mixture model to identify different running patterns (considering 5 km intervals) and their distribution across ages and gender. This information can help runners to train and run more intelligently. This method is also able to provide accurate predictions for the final arrival time from intermediate time records, which are comparable to state-of-the-art approaches.

Regarding the applicability of our approach, we remark that the idea of comparing group density distributions fairly within a single model is an attractive research path, that could result in a huge and broad number of applications. It can be applied to many problems involving stratified data and a certain control variable (e.g., age, gender, nationality). In problems concerning group data or any competitive human activity, sharing the mixture weights across groups is a sensible assumption. Some application examples can be found in pediatrics (e.g., comparison of children population according to weight and height), social sciences (e.g., analysis of gender impact on actual salary income across countries), or pharmaceutics (e.g., monitoring certain drug responses according to some patient covariates).

## Supporting Information

S1 Marathon Database File Data used in this study as a.mat file. Further descriptions are found in the README files inside.
(MAT)
